# Existing contradictions and suggestions: flipped classroom in radiology courses of musculoskeletal disease under Chinese medical educational mode from medical imaging student perspective

**DOI:** 10.1186/s12909-020-1991-2

**Published:** 2020-03-17

**Authors:** Su Wu, Shinong Pan, Ying Ren, Hong Yu, Qi Chen, Zhaoyu Liu, Qiyong Guo

**Affiliations:** 1 Department of Radiology, Shengjing Hospital of China Medical University, Shenyang, China; 2 Medical imaging, China Medical University, Shenyang, China; 3 Office of Educational Administration, Shengjing Hospital of China Medical University, Shenyang, China

**Keywords:** Flipped classroom, Radiology education, CTDI-CV, Critical thinking, Undergraduate education

## Abstract

**Background:**

Flipped classrooms have already begun to be used in many universities aboard, and they now make up for some of the short comings of the traditional classroom. We introduced the concept of flipped classrooms into a radiology class in China and evaluated the students’ performance to find out whether it was a better learning method. Furthermore, we have attempted to identify the problems of application of flipped classrooms (as practiced under the Chinese education system) and make suggestions.

**Methods:**

Facilities made videos and prepared clinical cases and short lectures for the flipped classroom. A total of 55 undergraduate radiology students were asked to finish pre-class learning and pre-learning assessment, participate in a flipped classroom about bone malignant tumours, and complete questionnaires. Teachers were also need to finish the survey.

**Results:**

1. The students showed good performances in the pre-learning assessment. The mean scores for three pre-learning assessment were 89.77, 96.54, and 93.71, respectively; the median scores were 90, 97.5, and 94, respectively.
2. After they attended the flipped classroom, their mastery of knowledge (case-solving skills, basic feature command, comparison ability, and overall knowledge command) showed improvements; after flipped classroom, the scores for these knowledge factors improved to 81.25, 85.42, 85.42, and 85.42%, respectively, compared to the scores they obtained before taking the flipped classroom (1.25, 68.75, 64.58, and 72.92% respectively).
3. The students’ discussion time and student-teacher-communication time increased, and the students’ questions were solved satisfactorily.
4. CTDI-CV showed no improvement in critical thinking skills after taking the course.
5. The time spent in previewing (pre-class video watching, material reading, and pre-learning assessment) increased significantly.

**Conclusions:**

Flipped classrooms, when tested in a radiology classroom setting, show many advantages, making up for some inadequacies of didactic classrooms. They provide students with better learning experiences. We can continue to practice flipped classroom methods under the curriculum, but we still need to make improvements to make it more suitable for the Chinese medical education mode.

## Background

Modern Chinese college and university teaching methods are mainly didactic due to their comprehensive use of resources. It uses a minimum number of teachers to convey a large amount of information to a great number of students [1, 2]. In medical school, this method helps students to learn both foundation courses and professional courses within lesser teaching time. However, as research shows, it also has drawbacks; Considering the attention spans of medical students [3], the lecture time should be around 30 min, hours of lectures can bore students easily and reduce learning efficiency. Furthermore, the traditional didactic teaching mode causes students to lack independent learning ability, problem solving skills, and critical thinking skills, which are important to life-long learning and clinical practice [4]. This causes a huge gap between theoretical knowledge and clinical trials when Chinese medical students begin their internships and find that they know nothing about diagnosis and treatment.

Flipped classroom has been promoted in recent years, and it requires pre-learning for students, group discussion, and teacher-student interactions in class [5]. Moreover, adding online modules (videos, online exercise systems, visual reality, etc.) to teaching contents benefits students’ performance [6–8]. It changes memory-based learning into collaborative and application-oriented learning [9]. Instead of learning through lectures, students learn those materials before class at their own paces, and instructors help students to consolidate knowledge, thus combining knowledge with practice in class [10]. During various activities, students’ motivation to learn was provoked, and they gained positive experiences through learning [11]; their critical thinking skills, analysis skills, communication skills, and cooperative working ability improved [12]. Teachers can better understand what students need and provide feedback immediately. Instructors also have more autonomy and flexibility to select teaching materials and increase teaching depth [13].

Flipped classrooms have been applied in many medical schools in the United States, and students showed improvements in many ways. The application of radiology courses also achieved great success [14–16]. Meanwhile, Chinese medical education needs to keep up with the times, improve the medical curriculum setting and teaching methodology, and pay more attention to clinical training [17]. The aim of our study was to apply the flipping method in Chinese radiology courses and try to modify the model to better adapt it to China’s teaching system, and provide suggestions.

## Methods

**Fig. 1:**
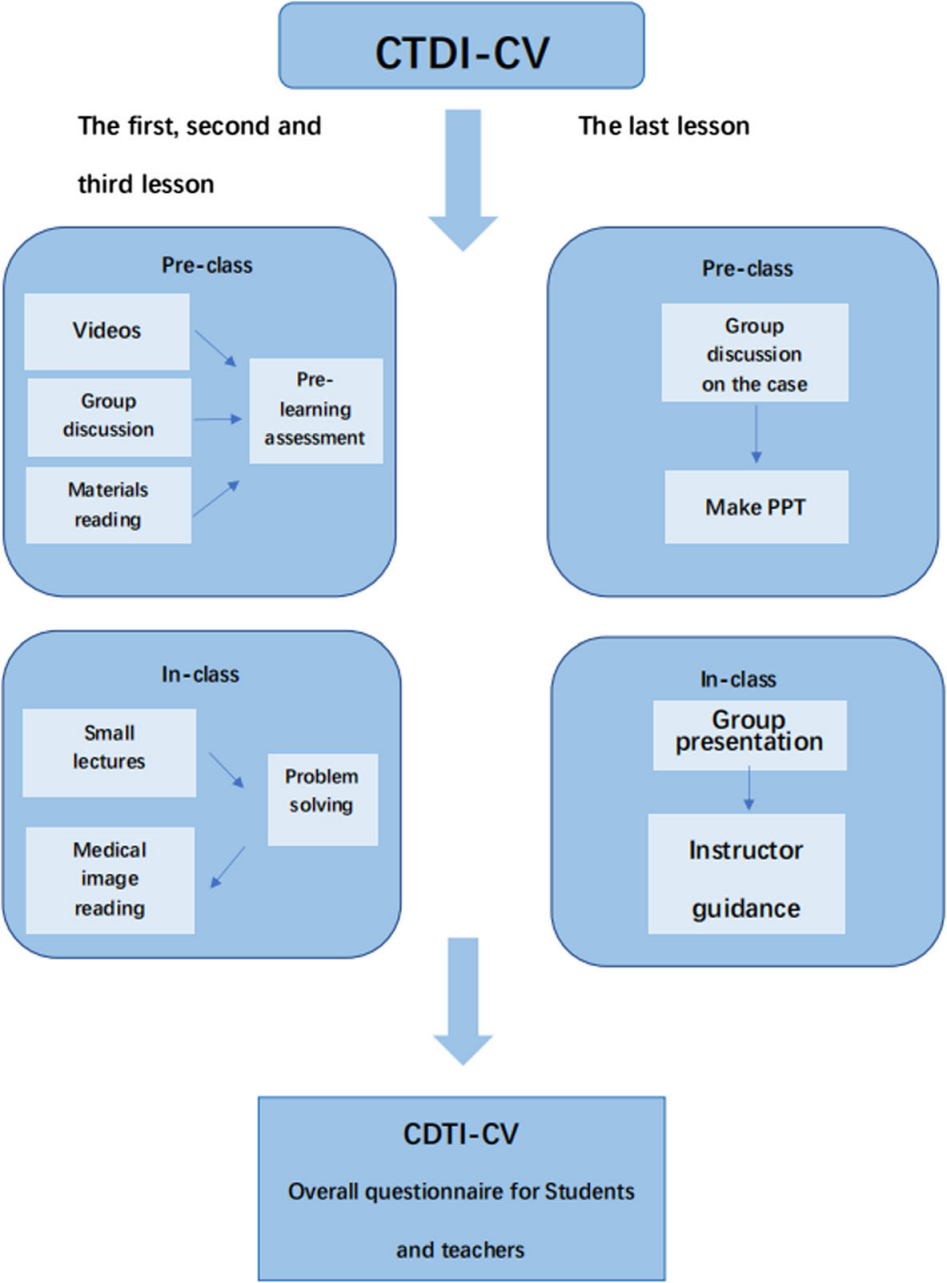
Flowchart of flipped classroom

The 55 participants were radiology students in their fourth grade and were divided into 5 groups. Ethical approval was received by Ethics Committee of Shengjing Hospital of China Medical University. The Ethics Code is 2018PS476K. All potential participants were informed about the purpose and content of the study. The written informed consent was obtained from all participants. The curriculum was based on Practice of Radiology, 3rd edition, which includes malignant bone tumour, bone developmental disorders, and tumour-like bone disorders. The teaching administration allotted 4 lessons in 2 weeks for the flipping method, each flipped classroom having 2 h. There were two teaching assistants and one instructor. This is the second time we designed a flipped classroom. Figure 1 is the flow chart of flipped classroom.

### Pre-class

Pre-class materials for the first three lessons included videos, teaching materials (Practice of Radiology, 3rd edition) and pre-learning assessment. Each video was 10 to 30 min long, including imaging features of diseases and typical radiological images. Those videos could be downloaded from the website. Teaching assistants allotted pre-class materials to students 1 month before the first flipping class so that they could be well prepared. Considering students need to prepare for their final examinations, early allotment of materials allows students to have sufficient time to learn by themselves. Students were asked to watch videos, read the relevant parts of the book, At the same time, we informed all the students the phone numbers, WeChat accounts, and email addresses of the instructor and teaching assistants. The teachers and students were in the same WeChat group so that they can communicate easily and openly. Students asked and discussed questions in the group. Teachers regularly encouraged students to work hard, complete assignments on time, and answered questions in the WeChat group and sometimes shared some reading materials with the students.

The day before each flipping class, teaching assistants allotted examination papers, which contained 10 Q&A questions that were designed especially for each class. Before the class began, teaching assistants collected those papers and graded them.

Pre-class materials for the last lesson were five cases and one complex case selected by the instructor. Medical backgrounds, laboratory examinations, and radiology images of patients were provided. Each group had one case to discuss, and they were required to make a PPT about image description, diagnosis, and differential diagnoses regarding their case. Everyone was required to prepare the complex case for discussion in class.

### In class

The first three lessons had three parts. In the first part, the teacher made short lectures about the knowledge that was not included in the videos; this part helped students replenish their knowledge. In the second part, the instructor answered the students’ questions regarding their pre-class learning. The third part was medical image reading. Last time, when we used Multi-disciplinary Team (MDT) mode and invited pathology teachers to teach the development of bones and cytology of bone tumours, the students reflected that it was not very helpful for theoretical learning. This time we changed the subject into medical image reading; before every class, the instructor selected representative medical cases from PACS system, the teacher made demonstrations regarding image description, and then the students practiced under the teacher’s instruction in class.

In the last lesson, each group elected representatives to show their PPT, which allowed students to put their knowledge to use and enhance their problem-solving skills. The instructor corrected wrongly comprehended information and made comments after each presentation. Then the whole class discussed the complex case, and the instructor revealed the answer and made conclusions at the end of the class.

### After class

Because there were conflicts between the course’s timing and final examination review, a post-test was not designed. After all the flipped classes were completed, students were asked to finish a questionnaire. The instructor and two teaching assistants were also asked to fill a survey.

### Data analyze

The pre-class examination scores were analysed with Excel. The mean score and median score were calculated to show the average level of mastery.

In order to study the impact of flipped classes on critical thinking, students were asked to finish the Chinese version of critical thinking disposition inventory (CTDI-CV) before the first flipped classroom began and after the fourth flipped classroom was completed [18]. CTDI-CV contains seven parts, which are truth seeking, open-mindedness, analyticity, systematicity, critical thinking self-confidence, inquisitiveness, and cognitive maturity. The total score of CTDI-CV was 70 ~ 420; less than 210 means negative critical thinking skills, 211 ~ 279 shows that the result is unclear, 280 ~ 350 means positive critical thinking skills, and more than 350 indicates strongly positive critical thinking skills. The total score of every trait was 10 ~ 60; less than 30 means negative critical thinking skills, 31 ~ 39 means the result is unclear, 40 ~ 50 means positive critical thinking skills, and more than 50 indicates strongly positive critical thinking skills. CTDI-CV data were collected and presented in mean ± standard deviation. SPSS was used to analyse internal consistency reliability. To evaluate critical thinking improvement after taking flipping class, two-sample *t*-tests were used.

We designed a questionnaire for students, and it includes their degree of mastery of knowledge before and after taking the flipped class, their opinions about the class schedule, and the time of preview. We also designed a questionnaire for teachers regarding their attitude towards this course. Both questionnaires were collected and analysed.

## Results

### Pre-class

**Fig. 2:**
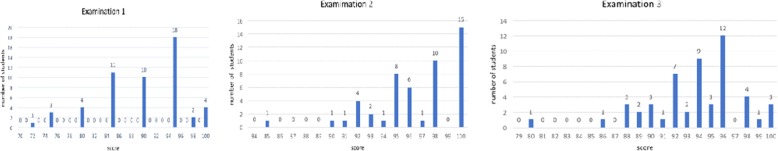
Histogram of pre-learning assessment score

Table 1 is the scores of three pre-learning assessments. Figure 2 is the histogram of three pre-learning assessments.

**Table 1:** The score of pre-learning assessment

	Examination1	Examination2	Examination3
Average score	89.78	96.54	93.71
Minimum-maximum	72–100	85–100	80–100
Median	90	97.5	94

Table 2 is the results of the pre-class section in overall questionnaire.

**Table 2:** Pre-class section in Overall questionnaire

	Flipping class	Traditional class	*t* value
Pre-class learning time (minute)	120 ± 67.88	21.15 ± 30.3	9.195
Pre-class discussion time (minute)	33.33 ± 28.63	17.61 ± 23.61	2.898

Table 3. CTDI-CV scores before flipped classroom.

**Table 3:** CTDI-CV scores before flipped classroom

Critical thinking traits (first)	Score	negative	Unclear	Positive	Strongly positive
**truth seeking**	39.89 ± 5.64	0	22	23	2
**open-mindedness**	42.28 ± 5.97	1	12	29	5
**analyticity**	43.40 ± 7.26	3	11	28	5
**systematicity**	33.04 ± 2.84	7	39	1	0
**critical thinking self-confidence**	38.79 ± 9.07	7	20	17	3
**inquisitiveness**	43.68 ± 8.40	4	10	23	10
**cognitive maturity**	42.83 ± 6.70	1	14	25	7
**overall**	283.91 ± 29.83	1	19	26	1

**Fig. 3:**
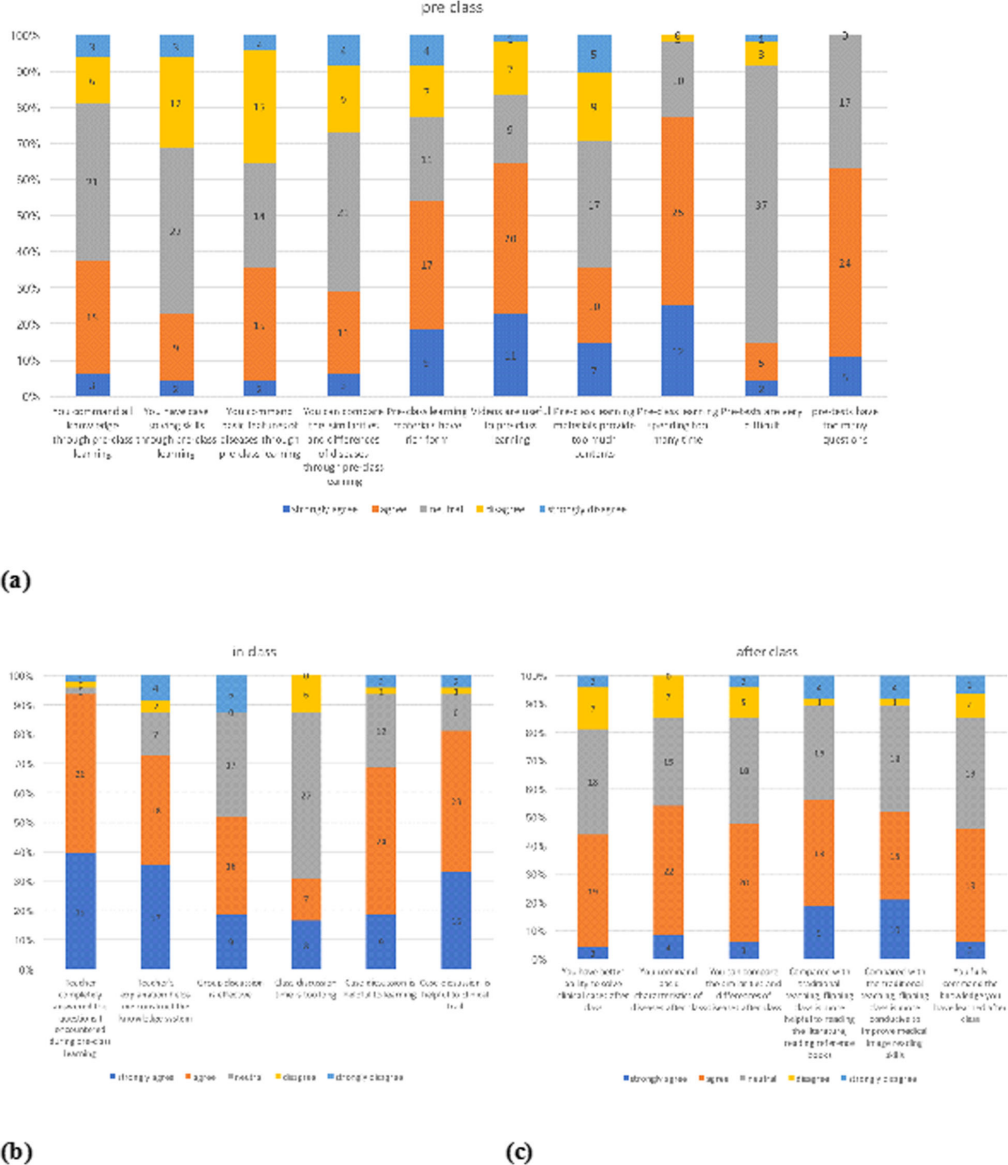
Overall questionnaire for students

Regarding the aspect of overall knowledge command, case-solving skills, basic features of diseases command, and comparison of different diseases, 81.25% (39), 68.75% (33), 64.58% (31), and 72.92% (35), respectively, of the students mastered more than half of the knowledge (Fig. 3a). This indicates that the students gained a relatively good command of knowledge through pre-class learning.

A total of 54.17% of students thought that the pre-class materials had variety in terms of forms, and 64.58% (31) of students thought the videos were useful, though 18.75% (9) were neutral about it. Adding short videos in materials can stimulate students’ enthusiasm and uploading videos on the website allows them to watch them at any time (Fig. 3a).

We divided the students into 5 groups, with each group having a WeChat group where they could have both online and offline discussions After grouping and the assigning of online discussion groups and tasks, students spent more time in exchanging ideas and solving problems. There is a significant difference between the flipped classroom and the traditional classroom in terms of pre-class learning time and discussion time. (Table 2).

However, almost 80% of students thought pre-class learning was taking up too much time (77.08%, 37); Students who thought that the materials had many contents and felt that these contents were appropriate made up 35.42, and 63.04% (29) of students thought the pre-learning assessment had too many questions.

### In class

In China, most universities and colleges do not have office hours or teaching assistants, so students can hardly find someone who can solve their problems. In our flipped classrooms, we designed problem-solving components, where students could ask questions that they found during self-study. This component was welcomed by students, and more than 90% of students said that their questions were answered. Small lectures also showed great effects, with 72.92% of students thinking that small lectures were useful. Compared with lectures lasting many hours, students could fully concentrate on the topic and therefore had higher efficiency. When designing small lectures, teachers extracted key contents as much as possible, and they then explained it in a straightforward way to guarantee easy understanding for students. The medical image reading component was also highly recognised; 68.75 and 81.25% of students thought it helped them learn theoretical knowledge and solve clinical problems, respectively (Fig. 3b).

About half of the students thought group discussion was effective (52.08%), while 35.42% (17) were neutral, and 12.5% (6) thought it was a waste of time. A total of 31.25% (15) of students thought class-discussion time was too long for the class, and 56.25% thought it was appropriate. This shows that, while more students participated in group discussion, some were still not involved (Fig. 3b). This is largely due to the inadequate preparation of the students, because of which they were unable to express their views during the discussion or could not keep pace with others.

### After-class

Table 4 is the CTDI-CV scores after flipped classroom. Table 5 is the internal consistency (alpha) of two CTDI-CV questionnaires.

**Table 4:** CTDI-CV scores afteThe 55 participants were radiology students in their fourth flipped classroom

Critical thinking traits (second)	Score	negative	Unclear	Positive	Strongly positive
**truth seeking**	37.44 ± 8.07	11	19	16	4
**open-mindedness**	41.04 ± 8.42	4	18	22	6
**analyticity**	40.86 ± 7.88	4	21	19	6
**systematicity**	33.94 ± 3.24	9	38	3	0
**critical thinking self-confidence**	38.54 ± 6.52	6	22	20	2
**inquisitiveness**	42.82 ± 7.19	1	19	21	9
**cognitive maturity**	40.00 ± 9.45	11	9	26	4
**overall**	274.64 ± 31.22	0	30	20	0

**Table 5:** Internal consistency (alpha) of two CTDI-CV questionnaires

Critical thinking traits	Internal consistency (alpha)	
	First CTDI-CV (***n*** = 47)	Second CTDI-CV (***n*** = 50)
**truth seeking**	0.416	0.756
**open-mindedness**	0.241	0.650
**analyticity**	0.264	0.239
**systematicity**	0.286	0.639
**critical thinking self-confidence**	0.748	0.698
**inquisitiveness**	0.635	0.591
**cognitive maturity**	0.654	0.806
**overall**	0.748	0.887

The first CTDI-CV overall score was 283.91 ± 29.83 (Table 3), which indicates that students have positive critical thinking skills, and the second CTDI-CV overall score was 274.64 ± 31.22 (Table 4), which means the result is not clear. After completing the flipped classroom, scores on systematicity improved. In both tests, students had positive critical thinking skills in open-mindedness, analyticity, inquisitiveness, and cognitive maturity; the result was unclear in truth seeking, systematicity, and critical thinking self-confidence. Homogeneity of variance test [F = 1.095, F < F_a/2, (v1, v2)_ (a = 0.05, F_a/2, (v1, v2)_ = 1.60)] showed that the overall variance was homogenous. Two-sample *t*-test was used to observe whether there was a statistical difference between the two CTDI-CV statistics. The difference between the two groups of data was not statistically significant, and there was no improvement of critical thinking skills after taking the flipped class (Table 6).

**Table 6:** Internal consistency (alpha) of two CTDI-CV questionnaires

Critical thinking traits	First CTDI-CV (***n*** = 47)	SecondCTDI-CV (***n*** = 50)	***t*** value
**truth seeking**	39.89 ± 5.64	37.44 ± 8.07	1.72
**open-mindedness**	42.28 ± 5.97	41.04 ± 8.42	0.83
**analyticity**	43.40 ± 7.26	40.86 ± 7.88	1.65
**systematicity**	33.04 ± 2.84	33.94 ± 3.24	1.45
**critical thinking self-confidence**	38.79 ± 9.07	38.54 ± 6.52	0.16
**inquisitiveness**	43.68 ± 8.40	42.82 ± 7.19	0.54
**cognitive maturity**	42.83 ± 6.70	40.00 ± 9.45	1.69
**overall**	283.91 ± 29.83	274.64 ± 31.22	1.49

**Fig. 4:**
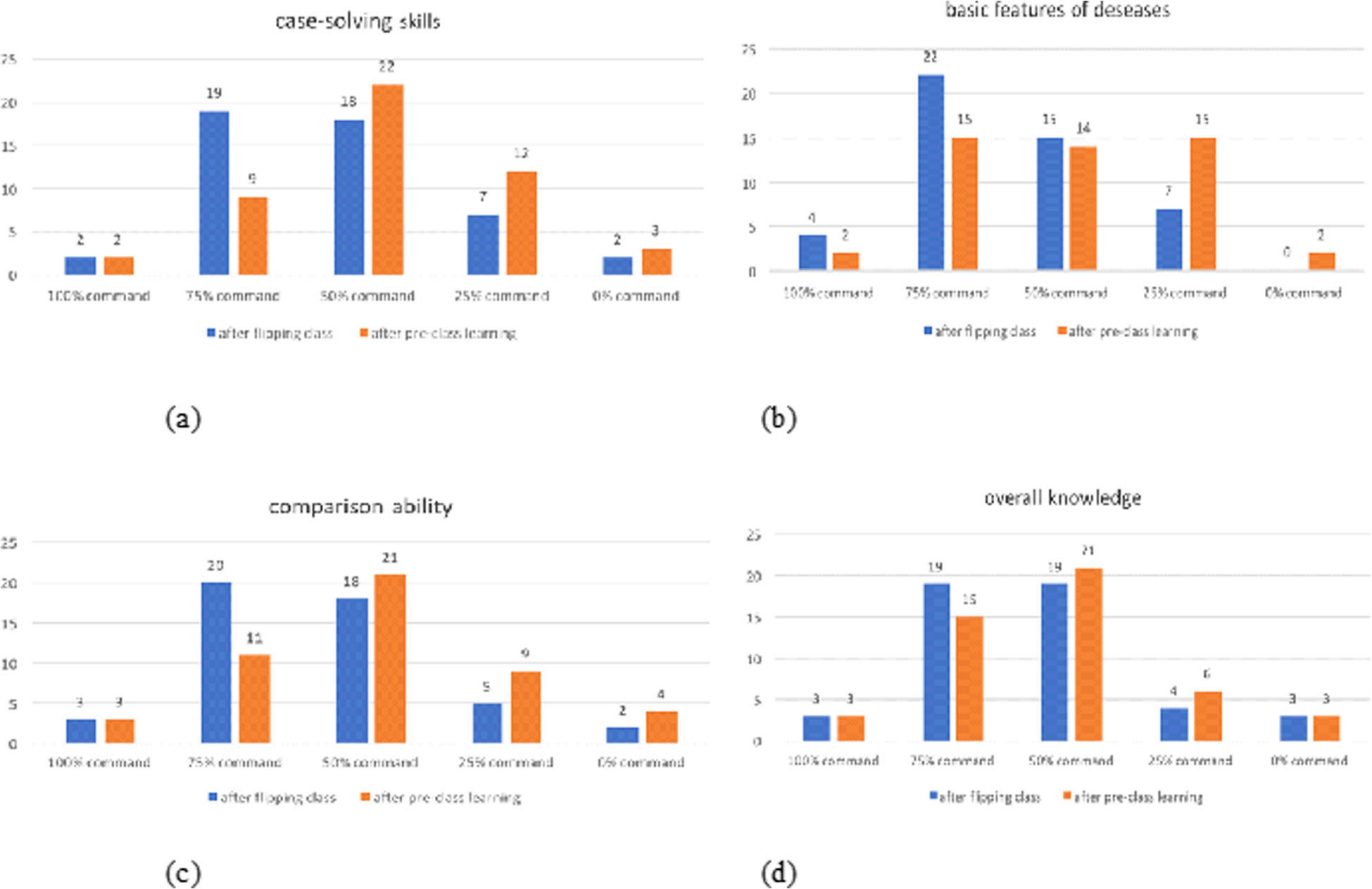
Comparison of mastery of knowledge between ‘after pre-class learning’ and ‘after flipped class’

We analysed the same aspect of learning result (overall knowledge command, case-solving skills, basic features of diseases command, and comparison of different diseases); 81.25, 85.42, 85.42, and 85.42% of students, respectively, had mastered more than half of the knowledge (Fig. 3c). Compared to the results before pre-learning calculation (81.25, 68.75, 64.58, 72.92%), the mastery of knowledge was elevated. Compared with traditional teaching, 56.25 and 52.08% of students thought that the flipped class was more helpful for material reading and clinical skills improvement (Fig. 4).

### Overall

**Fig. 5:**
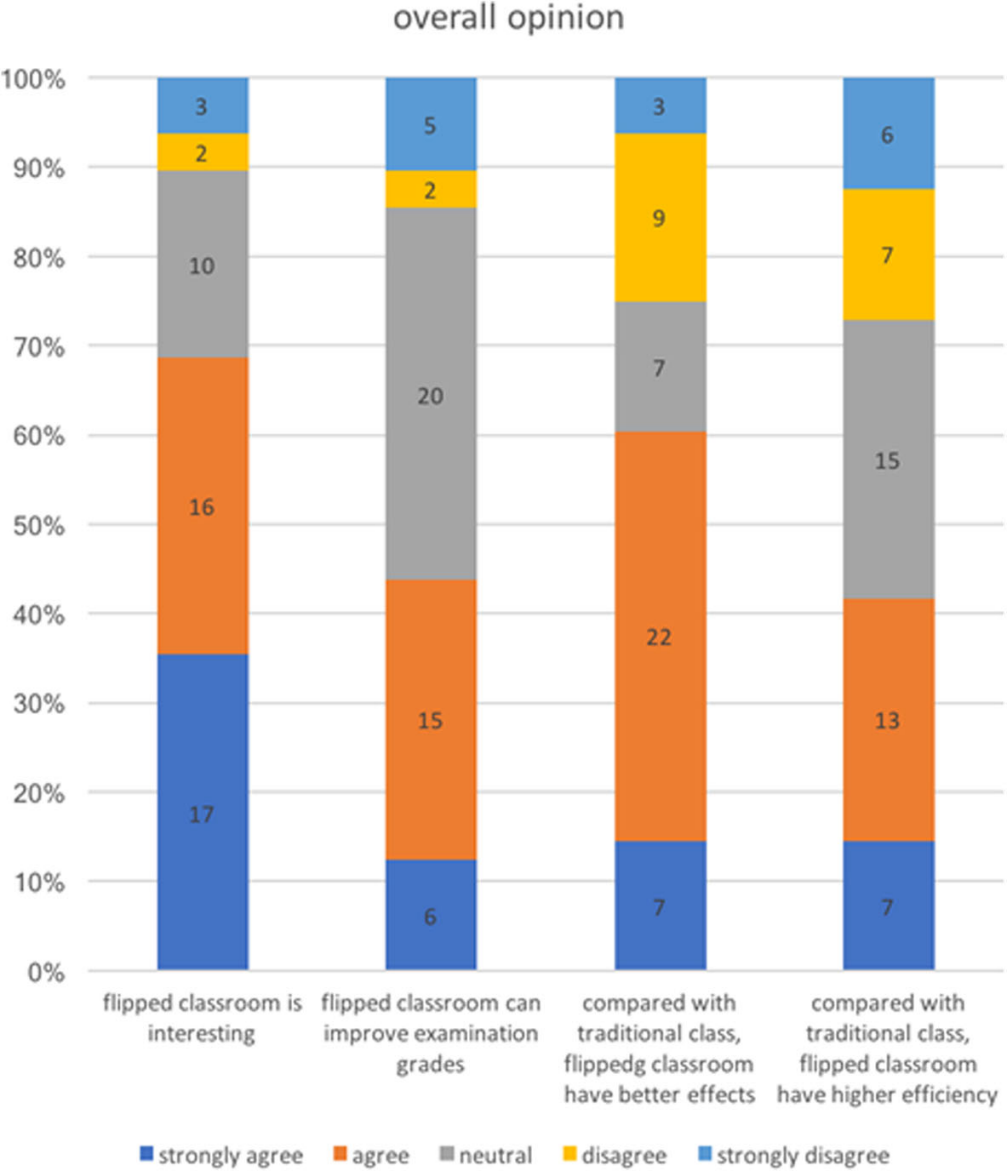
Overall opinion on flipped classroom

A total of 26 students (55.32%) thought the contents were appropriate and reasonable, and they were interested and took the initiative to participate in interactive activities. A total of 8 students thought that there was not much difference between taking a flipped class and didactic class, and 9 students did not want to participate. One student made the comment that hardly anything could be learned through the flipped class, and one said that he had some interest in it. Flipped classes are diverse in terms of forms of modules; 68.75% (33) of students thought flipping was interesting, and 60.42% (22) of students thought that the flipped class had a greater effect on their study than the didactic method (Fig. 5). A total of 36.17% of the students hoped to adopt the flipped mode for part of the chapter, and 29.79% of students wanted to apply case-study (mode of the last lesson) into the last lesson of each module. A total of 17.02% of students did not want to apply the flipping method into the class, 14.89% of students wanted to apply it into the whole radiology lesson, and one did not care. Students also wanted to add the PPT used in class and other teaching plans into the pre-class materials.

The survey for the instructor and two teaching assistants shows that the average time spent on the flipped classroom was 6 h, and time spent on the traditional class was 2 h. While preparing the course, the two teaching assistants checked books and PACS systems in both the flipped classroom and the traditional classroom modes. The instructor checked books, papers, PACS systems, and foreign universities’ teaching videos. Three faculty members thought that the degree of knowledge acceptance of students in the flipped classroom was good and that their acceptance in the traditional class was generally good. They wanted to apply the flipped classroom in some of their courses.

We also conducted a survey among the teachers who came to listen to the flipped classroom. Five teachers filled the questionnaire. Regarding the aspects of students’ performance and class design, they all thought that it was good: flipped classrooms should be used in some parts of the class. However, only two out of five teachers were willing to apply the flipped classroom approach, two did not want to participate, and one did not show a positive attitude. Teachers thought that the flipped classroom helps to establish learning initiative and consciousness and improves class participation. The current existing problems are 1) how to unify the flipped classroom approach and examination preparation of students and 2) how to increase the depth of people’s knowledge regarding flipped classrooms.

## Discussion

### Advantage

This is the second time we introduced the ‘flipping’ method into a radiology class; this time, we considered our previous study participant students’ opinions and made several modifications [19]. We allotted pre-class material earlier this time because, last time, the timing of the course was close to the final examination period and, consequently, students did not participate in the previous flipped classroom study. We considered that it would thus take more time for students to find the best way to learn by themselves. Another modification was replacing MDT with medical image description. MDT requires good mastery of multidisciplinary knowledge. However, undergraduate students find it difficult to meet this requirement. Medical image description is more realistic for the undergraduate level and helps students to apply their knowledge. These two modifications have improved the results of the current flipped classroom. The results of our surveys are similar to those of Zeng Rui et al [20]. Through pre-learning (including watching online-videos, group discussions, and material reading), most of the students were able to gain a good command of basic knowledges (mean scores: 89.77, 96.54, and 93.71, median scores: 90, 97.5, and 94). After taking the flipped classroom, their mastery of knowledge showed improvements. A total of 90% of students said that their questions were solved satisfactorily, 72.92% of students thought that small lectures were useful, and 68.75 and 81.25% of students thought that the medical image reading section helped them to learn theoretical knowledge and solve clinical problems, respectively. This reflects the students’ recognition of the flipped classroom and the good teaching effect of flipped classroom.

Compared with didactic teaching modes, flipped classrooms create an environment for self-learning and increase students’ learning motivation [21]. Besides this, the interaction between instructors and students is also enhanced. After the pre-learning section, instructors and students have more time for discussion in the classroom, the exchange of ideas, and collaborative problem solving, thus improving teaching effectiveness. Furthermore, this improved the overall quality of students’ learning and improved their ability to study independently, find problems, and solve problems.

### Limitations

There are several limitations in this survey. First, we used CTDI-CV to determine whether there is an improvement in students’ critical thinking skills, but this may be too general for a radiology class; furthermore, a few classes will not have a great influence on students’ thinking modes, and the course is thus unlikely to improve students’ thinking ability. The second limitation is the pre-learning assessment section; 10 Q&A questions require a long time to answer, and teachers’ ratings are subjective. As a result, the degree of mastery of knowledge may have deviated from reality. We think pre-learning assessment should use some multiple-choice questions from question banks to save students’ time and guarantee objectivity. Third, there are no after class tests in the study. This component could help students consolidate knowledge, thus helping us to understand the mastery level of students and, consequently, evaluate the true effect of the ‘flipping’ approach. Judging the effect of learning through the feelings of students is subjective. Forth, the students’ thinking mode is established through many years of study, and they had already become used to the teacher’s method of instilling knowledge; therefore, it is unrealistic to change the mode of learning through several flipped class.

### Contradictions and suggestions

There are four major contradictions between the flipped classroom and the current teaching model. Flipped classroom requires changes in both learning mode and teaching mode as well as the evaluation system of students.

1. Passive teaching into autonomous learning: In traditional didactic modes, students master knowledge structures and key points in lectures and then read books targeted toward them after class. On the contrary, flipping class requires them to learn by themselves. For most Chinese students, who do not have the ability to quickly read books and dissertations, this will increase a lot of reading tasks considerably. It is thus natural for some students to not participate in it because they do not want to accommodate this mode. Increasing document discussion classes and encouraging students to complete small projects will help them to improve their ability to learn independently.
2. A more complete student evaluation system: Many students have stated that flipped classrooms do not improve their final examination grades because nowadays the evaluation system estimates students’ mastery of knowledge based on only one theoretical examination; therefore, by simply memorizing the key points of the book and question bank, students are able to obtain good grades. If class performance and clinical performance were added into the evaluation system, more students would want to take flipped classroom courses, and this would be helpful for the cultivation of their abilities.
3. Balance between clinical works and less preparation time: Flipped classrooms contain many different components and teacher-student interactions, so global control is needed. Furthermore, how to give clear explanations within a short period (short videos and short lectures) is a big challenge. Preparation time for flipped classrooms is three times higher than that of traditional classrooms, while most teachers need to focus on clinical trials, conducting research, and engaging in teaching activities [22]; it is difficult for them to devote so much time to preparing flipped classrooms. This has also led to the reluctance of teachers to conduct flipped classrooms.
4. Flipped classroom is an exploratory course in our country. The student spent too much time before flipped class was an issue. After many discussions between us, the teaching managers and the course coordinators from our college and hospital, we decided not to make any adjustments at timetable for now, and just followed the regular teaching schedule. In the future, we will put down the burden of the students by definitely increasing the class hours to alleviate the time pressure when we carry out this course. Meanwhile, we will continue to think about how to adjust the timetable and class hours in our next flipped classroom.

## Conclusions

Overall, the flipped classroom approach showed great advantages in the radiology class setting; it made up for some of the inadequacies of didactic classrooms and gave students a better learning experience. However, due to the abundant time it took up, students thought it was not an effective way to learn and that it would not be able to improve final examination grades. So, we still need to make improvements to make such classrooms more suitable for the Chinese teaching mode and we will continue to promote flipped classrooms in the radiology class setting and try to expand its application.

## Data Availability

The data and materials used and analysed during the study are available from the corresponding author on reasonable request.

## Abbreviations

CDTI: CV Chinese version of critical thinking disposition inventory
MDT: Multi-disciplinary Team
